# Association of Advanced Glycation End Products with Diabetic Retinopathy Severity in Lithuanian Patients

**DOI:** 10.3390/medicina61111956

**Published:** 2025-10-30

**Authors:** Aiste Varoniukaite, Ugne Poskiene, Deimante Paskeviciene, Diana Simoniene, Lina Radzeviciene, Rasa Verkauskiene, Vilma Jurate Balciuniene

**Affiliations:** 1 Medical Academy, Lithuanian University of Health Sciences, Eiveniu 2, LT-50161 Kaunas, Lithuania; 2 Institute of Endocrinology, Lithuanian University of Health Sciences, Eiveniu 2, LT-50161 Kaunas, Lithuania

**Keywords:** diabetic retinopathy, AGEs, diabetes mellitus

## Abstract

*Background and Objectives*: Diabetic retinopathy (DR) is a major microvascular complication of diabetes mellitus (DM) and a leading cause of blindness among working-age adults. Advanced glycation end products (AGEs) contribute to DR pathogenesis through oxidative stress and inflammation. This study aimed to evaluate the association between AGE levels and DR severity in Lithuanian patients with type 1 (T1D) and type 2 (T2D) diabetes. *Materials and Methods*: An observational cross-sectional study enrolled 152 patients with T1D and T2D from a tertiary university hospital. AGE values were measured with a non-invasive AGE Reader device. DR severity was assessed through ophthalmological examination. *Results*: The overall DR prevalence was 54.6%. AGE values increased with the severity of DR, though not significantly between the DR groups (*p* = 0.386). The mean AGE value was higher in T2D compared with the T1D group (2.429 vs. 2.217, *p* = 0.011). In T1D, AGE values were higher in the advanced DR group compared with the non-DR patients (2.445 vs. 1.905, *p* = 0.007), whereas no significant difference was found in the T2D subgroups. In T1D, AGEs correlated positively with both DM duration and higher HbA1c (*p* < 0.05). Patients with AGE values > 2.35 in T1D and 2.15 in T2D had an increased likelihood of having advanced DR. Patients in the non-DR group had significantly lower AGE values compared to those in the advanced DR group (OR = 0.118, 95% CI 0.025–0.566, *p* = 0.008). *Conclusions*: AGEs measured using skin autofluorescence may indicate DR severity in T1D but not in T2D and might not be a suitable standalone risk predictor. DM duration remains a significant determinant of advanced DR, highlighting the importance of early monitoring and timely intervention.

## 1. Introduction

Diabetic retinopathy (DR) is recognized as one of the most common complications of diabetes mellitus (DM). It is among the leading causes of blindness and visual impairment in working-age individuals in the Western world. Over 100 million individuals worldwide are affected by this disease [1,2].

DR is a significant inflammatory and neurovascular complication of DM, resulting from prolonged damage to the retinal capillaries and injury or dysfunction of the retinal neurons [3]. Key risk factors for developing DR include the duration of DM, hyperglycemia, dyslipidemia, microalbuminuria, and arterial hypertension (AH) [4]. Hyperglycemia contributes to increased oxidative stress in the retina, while retinal ischemia aggravates oxidative damage and triggers the release of proangiogenic factors, further advancing the disease [5].

Metabolic pathways such as the hexosaminidase pathway are disrupted by oxidative stress and are associated with increased advanced glycation end product (AGE) formation [6]. AGEs are formed through nonenzymatic reactions involving lipids, proteins, reducing sugars, or nucleic acids. When AGEs build up in bodily fluids, their abnormal concentration becomes toxic, contributing to various diabetic complications, retinal and optic nerve diseases, as well as Alzheimer’s and Parkinson’s disease pathogenesis [7].

In a sub-study of the Diabetes Control and Complications Trial, it was shown that skin AGEs are more reliable predictors of retinopathy than glycated hemoglobin (HbA1c), as they reflect hyperglycemia over a longer period [8,9,10]. However, AGE testing is not commonly used due to technical difficulties such as needing skin biopsy. Skin autofluorescence (AF) is a simple, quick, and non-invasive test for assessing AGE build-up in skin tissues that shows the characteristic fluorescence pattern of AGEs [9].

Skin AF may reflect AGE build-up in retinal vessels affected by DR and potentially serve as a predictor of DR severity, so the aim of this study was to evaluate the association of AGEs with DR severity in diabetes patients in a Lithuanian cohort.

## 2. Materials and Methods

### 2.1. Study Sample

The observational study included patients with type 1 (T1D) and type 2 (T2D) diabetes mellitus. Patients were recruited in the tertiary university hospital from regular outpatient and inpatient visits at the Department of Endocrinology of the Hospital of Lithuanian University of Health Sciences (LUHS) Kaunas Clinics in Kaunas, Lithuania that underwent full ophthalmological examination at the Department of Ophthalmology of the Hospital of LUHS Kaunas Clinics in Kaunas, Lithuania. The recruitment period was between 15 February 2022 and 15 August 2023. The patients who met the following criteria were included in the study: from 18 to 85 years of age; for T1D, the DM diagnosis was made more than 5 years ago; the ability to give written consent; no presence of dermatopathy; and an agreement to participate in all study examinations. The study procedures were in accordance with the ethical standards and approved by Kaunas Regional Ethics Committee of Biomedical Research, approval number BE-2-116. All procedures were carried out with ethical standards in accordance with the Declaration of Helsinki.

### 2.2. Ophthalmological Assessment

DR severity was evaluated by two experienced ophthalmologists based on full ophthalmological examination (including: best-corrected visual acuity using a Snellen chart, intraocular pressure assessed using air-puff tonometry, indirect ophthalmoscopy after pupil dilation with 1% tropicamide) and three 45° field color fundus images obtained using the Zeiss Visucam 500 (SN1100558, software version 5.1.0.0026, Carl Zeiss Meditec AG, Jena, Germany). Patients were graded to the following five DR groups according to the International Clinical Diabetic Retinopathy Disease Severity Scale [11]: no apparent DR (no microaneurysms or other DR lesions), mild non-proliferative DR (NPDR) (presence of only microaneurysms), moderate NPDR (presence of microaneurysms and other hemorrhages and/or hard exudates not meeting criteria for severe NPDR), severe NPDR (any of the “4-2-1” rule findings: ≥20 intraretinal hemorrhages in each of four quadrants, definite venous beading in two or more quadrants or prominent intraretinal microvascular abnormalities in one or more quadrants, and no signs of proliferative DR), proliferative DR (PDR) (presence of neovascularisation and/or vitreous or preretinal hemorrhage).

### 2.3. General Assessment

Medical history and current medications were collected through self-report interviews and accessible medical data. Anthropometric measures, including height in centimeters (cm) and weight in kilograms (kg), were obtained according to the World Health Organization guidelines [12]. Body mass index (BMI) was calculated as weight (kg)/height^2^ (m^2^). Blood pressure was measured at 5-minute intervals three times, and the average value was included in the study. The AH diagnosis was established regarding the previous medical history records and regular use of antihypertensive medication.

Blood samples included total cholesterol (TC), low-density lipoprotein cholesterol (LDL-C), high-density lipoprotein cholesterol (HDL-C) triglycerides (TG), and glycated hemoglobin (HbA1c). The albumin/creatinine (A/C) ratio was measured in urine. All samples were taken and evaluated in the certified laboratory of the Hospital of LUHS Kaunas Clinics.

AGEs were measured non-invasively using an AGE Reader device (DiagnOptics Technologies B.V., SN 00010604, Groningen, The Netherlands). Three measurements on the right forearm were taken for each subject by trained staff in a semi-dark room, and the mean numerical value was noted.

### 2.4. Statistical Analyses

Statistical analyses were performed using Statistical Package for Social Sciences 24.0 software (SPSS Inc., Chicago, IL, USA). Values are given as mean ± standard deviation (SD). The general characteristics of frequency and percentage were calculated; descriptive statistics were used to summarize all measurements. The normality of distribution was tested using the Kolmogorov–Smirnov test. Student’s (t) criterion was used for comparison of means for normal distributions, and the Mann–Whitney (U) test was used in skewed distributions. Differences between two means of independent samples were compared using Student’s *t* test. Categorical variables (presented as median values) were compared using χ^2^ or Fisher’s exact tests. Logistic regression models were used to assess the association of variables and DR severity. The receiver operating characteristic (ROC) curve was used to evaluate the efficacy of relevant indicators in DR severity groups; the optimal cutoff value for DR was estimated and chosen where the sensitivity and specificity was at maximum. *p* < 0.05 was considered statistically significant.

## 3. Results

A total of 152 patients, of which 69 were men (45.4%) and 83 (54.6%) were women with T1D (50.7%) and T2D (49.3%), were included in the analysis. The mean age was 51.85 ± 16.63 years (40.79 ± 14.40 for T1D and 63.20 ± 9.66 for T2D, *p* < 0.001). The mean duration of diabetes was 15.99 ± 11.18 years (20.27 ± 11.57 for T1D and 11.60 ± 8.89 for T2D, *p* < 0.001). The mean HbA1c was 8.34 ± 1.99% (8.30 ± 1.76 for T1D and 8.37 ± 2.21 for T2D, *p* = 0.814). The overall DR prevalence in the study group was 54.6%. Due to the low number of severe NPDR subgroup patients, further data analysis was based on three derivative DR groups: no DR (*n* = 69), mild-to-moderate NPDR (*n* = 56), and advanced DR as severe NPDR to PDR (*n* = 27).

The clinical characteristics of all patients and between DR severity groups are presented in Table 1. T1D, a longer duration of DR, a higher HDL-C, and a higher A/C ratio were more prevalent findings in the advanced DR group (*p* < 0.05), while a higher BMI was more prevalent in the non-DR group (*p* < 0.001). Other variables did not statistically significantly differ among the DR groups.

The mean value of AGEs assessed using skin autofluorescence was higher in T2D compared with the T1D group (2.429 vs. 2.217, *p* = 0.011). No differences in AGE values between the DR groups were observed (*p* = 0.08) in all study patients, though in the T1D subgroup the mean value of AGEs was higher in the advanced DR group compared to non-DR patients (2.445 vs. 1.905, *p* = 0.007), whereas no significant difference was found between T2D patients within different DR groups (*p* = 0.229).

While the AGE value tended to increase with increasing DR severity, intergroup differences failed to reach statistical significance (*p* = 0.386). In T1D, regarding the presence or absence of DR, the mean AGE value was significantly higher in those patients with a DR diagnosis (NPDR and PDR) vs. no DR diagnosis (2.326 ± 0.537 vs. 1.905 ± 0.543, *p* = 0.004).

The AGE value in all DM patients showed a significant positive correlation with age (r = 0.508, *p* < 0.001), the duration of DM (r = 0.301, *p* < 0.001), and creatinine concentration (r = 0.294, *p* < 0.001), while no significant correlation was observed with other variables. In a separate subgroup analysis, in both the T1D and T2D subgroups, significant correlations with AGEs included age (r = 0.525 and r = 0.488, respectively, *p* < 0.001) and the duration of DM (r = 0.497 and r = 0.296, respectively, *p* < 0.001). There was no significant correlation between HbA1c and AGEs (*p* = 0.206) in all DM patients, but in T1D the AGE value significantly increased with higher HbA1c values (r = 0.320, *p* = 0.005), but not significantly in the T2D subgroup (*p* = 0.310).

A logistic regression was performed to ascertain the effects of various parameters on the likelihood of advanced DR (severe NPDR to PDR). The duration of the DM was significantly associated with advanced DR (*p* = 0.001) in T1D, but not the T2D group. In the T2D group, a higher TC and A/C ratio were associated with advanced DR (*p* < 0.05). The higher AGE values were not a statistically significant factor in both T1D and T2D groups (Table 2).

**Figure 1:**
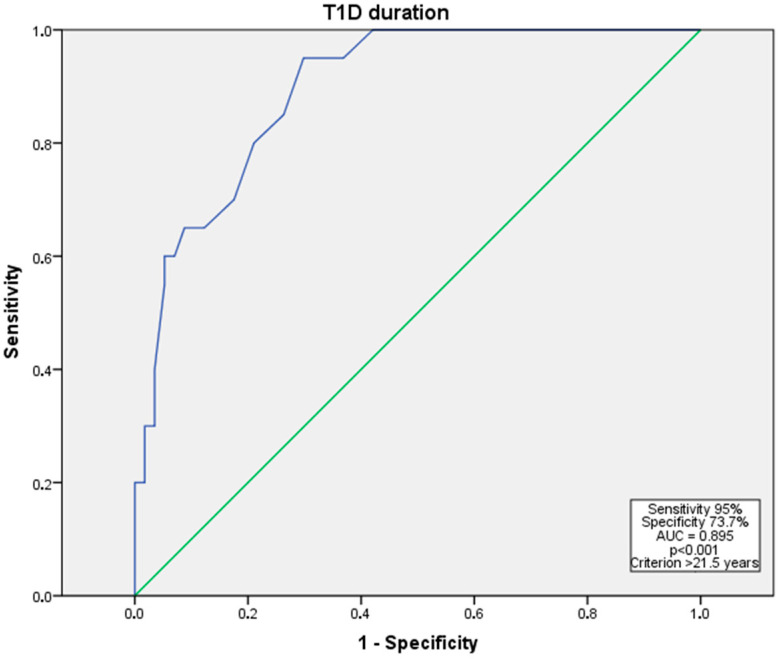
ROC curve for advanced DR (severe NPDR to PDR) risk in relation to diabetes duration years in T1D patient subgroup. Abbreviations: AUC—area under the curve; NPDR—non-proliferative diabetic retinopathy; PDR—proliferative diabetic retinopathy; ROC—receiver operating characteristic; T1D—type 1 diabetes mellitus. The blue line indicates the ROC curve, and the green diagonal line represents the reference line.

With a DM duration more than 21.5 years, the T1D subgroup patients had 5.4 times higher odds of having advanced DR (severe NPDR to PDR); the discriminative ability of the model was evaluated using the ROC test (95% sensitivity and 73.7% specificity, area under the curve (AUC) = 0.895, *p* < 0.001) (Figure 1).

The performance of TC and the A/C ratio as a predictor was poor in the ROC analysis (AUC = 0.469 and 0.361, respectively) and cannot be valued as a strong standalone variable.

**Figure 2:**
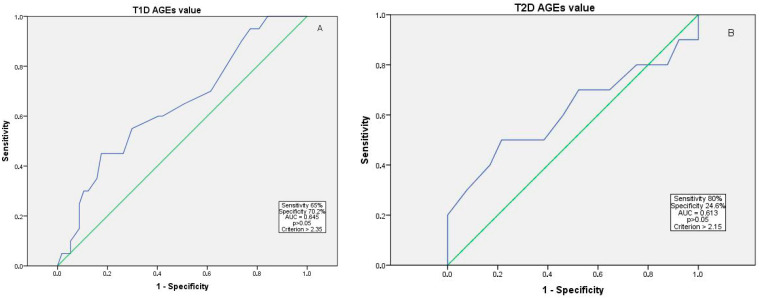
ROC curve for advanced DR (severe NPDR to PDR) risk in relation to AGE value in T1D (**A**) and T2D (**B**). Abbreviations: AGEs—advanced glycation end products; AUC—area under the curve; NPDR—non-proliferative diabetic retinopathy; PDR—proliferative diabetic retinopathy; ROC—receiver operating characteristic; T1D—type 1 diabetes mellitus; T2D—type 2 diabetes mellitus. The blue line indicates the ROC curve, and the green diagonal line represents the reference line.

AGE values greater than 2.35 in T1D and 2.15 in T2D were associated with an increased likelihood of having advanced DR, but the result was not statistically significant (65% sensitivity and 70.2% specificity, AUC = 0.645 for T1D, and 80% sensitivity and 24.6% specificity, AUC = 0.613 for T2D, *p* > 0.05) (Figure 2).

A multinomial logistic regression was conducted to examine the associations between clinical and biochemical variables and the severity of DR among all patients, including those with T1D and T2D (R2 = 0.497, *p* < 0.001). The DM duration was a significant predictor of DR severity across the groups, with each additional year of DM increasing the risk by 9.3% for the mild-to-moderate NPDR group compared to the non-DR group (OR = 1.093, 95% CI 1.034–1.155, *p* = 0.002) and by 21% for the advanced DR (severe NPDR to PDR) group (OR = 1.210, 95% CI 1.114–1.314, *p* < 0.001). A higher HDL-C (OR = 3.389, 95% CI 1.382–30.296, *p* = 0.008), higher TG (OR = 2.581, 95% CI 1.009–6.604, *p* = 0.047), and higher A/C ratio (OR = 1.022, 95% CI 1.003–1.041, *p* = 0.026) were significantly associated with a higher risk of advanced DR, but not the AGE value (*p* > 0.05). In the T1D subgroup analysis, where advanced DR served as the reference category, a higher AGE value was significantly associated with DR severity, as patients without DR had lower AGE values compared to those with advanced DR (OR = 0.118, 95% CI 0.025–0.566, *p* = 0.008). No other predictors, including the A/C ratio, AH, and dyslipidemia, were statistically significant (Table 3).

## 4. Discussion

Numerous individual studies and meta-analyses have explored various known, such as the duration of diabetes, hyperglycemia, arterial hypertension, dyslipidemia, etc. [13], and new risk factors for the progression of DR [14]; thus, some patients can develop DR and progress even without the contribution of well-known risk factors. Combined and new biomarkers are in need for individualized risk detection. Severe NPDR and PDR can lead to vision-threatening complications; therefore, the risk of progression to advanced stages of DR represents a critical factor in the search for novel predictors and personalized risk stratification models. AGEs play an important role in the pathogenetic mechanisms of DR [15] and few studies have found AGEs as an independent risk factor for DR [16,17]. A recent systematic review and meta-analysis from Martinez-Garcia et al. [18] indicated the use of skin AF as a sufficient diagnostic test even for DR screening in settings such as primary care centers.

Still, data from the Lithuania and Baltic region regarding the risk of advanced stages of DR in relation to AGEs alongside other established risk factors remain limited. Our results of 152 patients showed that the AGE value assessed using skin autofluorescence increased with the severity of DR, but the increase was not statistically significant between the DR groups in all study patients, though separately, in the T1D subgroup, the mean AGE value was significantly higher in the advanced DR (severe NPDR to PDR) group compared to non-DR patients. No such difference was observed in the T2D subgroup among the DR groups. In multinomial logistic regression models with different variables, the AGE value was significantly associated with the DR severity in the T1D subgroup, but not in the T2D subgroup model. Moreover, the AGE value in all DM patients positively correlated with age and the duration of DM calculated for the whole study population and separately for T1D and T2D subgroups, but only in the T1D subgroup were AGEs significantly correlated with HbA1c. These differences may be attributed to the longer duration of DM and prolonged exposure to hyperglycemia in patients with T1D compared to those with T2D, as prolonged hyperglycemia promotes the accumulation of AGEs, which localize intracellularly and activate multiple cellular signaling pathways leading to oxidative stress and the release of inflammatory factors contributing to microvascular complications [19,20,21]. Hence, Ying et al. [22] in a T2D large cohort provided evidence that AGEs are associated with both the prevalence and severity of DR independently of HbA1c, since AGEs reflect longer-term glycemic control and, according to Hirano et al. [8], may serve as a surrogate marker for the development of DR.

Although, in a univariate analysis of our T1D cohort, higher AGE levels were significantly associated with DR, a more comprehensive multivariate logistic regression model revealed that diabetes duration was the main risk factor for DR, rather than AGE levels. Although a multinomial logistic regression model identified the AGE value as a significant risk factor for advanced DR in the T1D subgroup, this association was not observed across the entire study population, including T2D patients. However, well-recognized risk factors such as the duration of diabetes remain among the strongest predictors of advanced DR. While the accumulation of AGEs is known to be associated with the natural aging process [23], our observation of higher AGEs in younger T1D patients compared with T2D patients suggests that other age-independent factors may contribute.

### Study Limitations

There are some limitations of this study. First, the study sample was relatively small, and the overall DR prevalence in the study group was 54.6%, which is higher than what was reported in previous studies, ranging from 20.8 to 39.6% [24,25,26]. A notably higher DR prevalence in our study could be attributed to the study design, as the recruitment process was in a tertiary university hospital, likely presenting with more severe or complex cases of our population. Second, the study population included only Caucasian patients; thus, the patient skin color could influence the AGE values. Third, diet and cooking preferences may influence the AGE value [27,28,29,30]; further research including eating patterns in the analysis should be performed.

## 5. Conclusions

AGEs measured using skin autofluorescence might have an association with the severity of DR in T1D, but not T2D, and might not be a suitable standalone risk factor for prognosing DR severity. More investigations and research regarding the association between AGEs and DR severity in both T1D and T2D should be performed. The impact of DM duration emphasizes the necessity of continuous monitoring to reduce the risk of DR progression with timely intervention.

## Figures and Tables

**Table 1:** The clinical characteristics of all patients and between diabetic retinopathy severity groups.

Variable	All (*n* = 152)	No DR (*n* = 69)	Mild-to-Moderate NPDR (*n* = 56)	Advanced DR (Severe NPDR to PDR) (*n* = 27)	*p* Value
Male, *n* (%)	69 (45.4)	29 (42.0)	26 (46.4)	14 (51.9)	0.672
T1D, *n* (%)	77 (50.7)	20 (29.0)	33 (58.9)	24 (88.9)	<0.001 *
Presence of AH, *n* (%)	89 (58.6)	44 (63.8)	33 (58.9)	12 (44.4)	0.224
Presence of dyslipidemia, *n* (%)	52 (34.2)	28 (40.6)	16 (28.6)	8 (29.6)	0.319
Smokers, *n* (%)	40 (26.3)	18 (26.1)	15 (26.8)	7 (25.9)	0.803
Age, years	51.85 ± 16.63	54.81 ± 15.50	50.66 ± 18.16	46.74 ± 15.05	0.08
Duration of DM, years	15.99 ± 11.18	9.67 ± 8.46	17.32 ± 7.87	29.41 ± 10.52	<0.001 *
SBP, mmHg	137.18 ± 18.58	136.96 ± 17.23	137.02 ± 20.02	138.11 ± 19.30	0.960
DBP, mmHg	83.96 ± 11.07	84.04 ± 9.98	84.30 ± 12.74	83.04 ± 10.33	0.886
HR, beats/min	80.80 ± 12.40	79.70 ± 10.31	81.23 ± 14.08	82.74 ± 13.72	0.531
BMI, kg/m^2^	29.86 ± 6.69	32.05 ± 6.84	28.96 ± 6.32	26.13 ± 4.95	<0.001 *
AGE value	2.32 ± 0.52	2.26 ± 0.51	2.37 ± 0.47	2.38 ± 0.63	0.386
HbA1c, %	8.34 ± 1.99	8.03 ± 1.98	8.54 ± 2.09	8.74 ± 1.71	0.204
TC, mmol/L	5.13 ± 1.44	5.37 ± 1.63	4.93 ± 1.09	4.96 ± 1.53	0.190
LDL-C, mmol/L	3.14 ± 1.00	3.26 ± 1.17	3.04 ± 0.90	3.06 ± 0.76	0.422
HDL-C, mmol/L	1.34 ± 0.37	1.26 ± 0.37	1.34 ± 0.31	1.54 ± 0.39	0.002 *
TG, mmol/L	2.03 ± 3.39	2.62 ± 4.79	1.61 ± 0.96	1.42 ± 1.77	0.152
Creatinine, μmol/L	75.14 ± 25.80	73.60 ± 24.33	78.94 ± 26.19	71.22 ± 28.52	0.355
A/C ratio, mg/mmol	8.24 ± 27.94	3.82 ± 11.13	6.09 ± 27.03	23.83 ± 48.42	0.005 *

* *p* value < 0.05. Data presented as mean ± SD or n (%). Abbreviations: A/C ratio—albumin to creatinine ratio; AGEs—advanced end glycation products; AH—arterial hypertension; BMI—body mass index; DBP—diastolic blood pressure; DM—diabetes mellitus; DR—diabetic retinopathy; HbA1c—glycated hemoglobin; HDL-C—high-density lipoprotein cholesterol; HR—heart rate; LDL-C—low-density lipoprotein cholesterol; NPDR—non-proliferative diabetic retinopathy; PDR—proliferative diabetic retinopathy; SBP—systolic blood pressure; TC—total cholesterol; TG—triglycerides; T1D—type 1 diabetes mellitus.

**Table 2:** Odds of advanced DR (severe NPDR to PDR) with various variables in a univariate logistic regression analysis.

Variable	T1D		T2D	
	*p* Value	OR [95% CI] for Severe NPDR to PDR	*p* Value	OR [95% CI] for Severe NPDR to PDR
Duration of the DM	0.003 *	0.668 [0.511–0.872]	0.082	0.874 [0.788–1.014]
AGE value	0.366	0.372 [0.044–3.73]	0.136	0.175 [0.018–1.727]
Total cholesterol	0.887	1.263 [0.050–31.909]	0.027 *	0.079 [0.008–0.745]
A/C ratio	0.355	0.986 [0.957–1.016]	0.023 *	0.965 [0.935–0.995]

* *p* value assessed by χ^2^ test < 0.05. Abbreviations: A/C ratio—albumin to creatinine ratio; AGEs—advanced end glycation products; CI—confidence interval; DM—diabetes mellitus; DR—diabetic retinopathy; NPDR—non-proliferative diabetic retinopathy; OR—odds ratio; PDR—proliferative diabetic retinopathy; T1D—type 1 diabetes mellitus; T2D—type 2 diabetes mellitus.

**Table 3:** AGE value in risk of advanced DR in multinomial logistic regression.

Comparison (vs. Advanced DR)	Predictor	Odds Ratio	95% CI (Lower–Upper)	*p* Value
No DR	AGE value	0.118	0.025–0.566	0.008 *
Mild-to-moderate NPDR	AGE value	0.587	0.208–1.656	0.314

* *p* value < 0.05. Abbreviations: AGEs—advanced end glycation products; CI—confidence interval; DR—diabetic retinopathy; NPDR—non-proliferative diabetic retinopathy.

## Data Availability

Data are unavailable due to privacy restrictions.

## Abbreviations

The following abbreviations are used in this manuscript:

A/C ratio	Albumin/creatinine ratio
AF	Autofluorescence
AGE	Advanced glycation end products
AH	Arterial hypertension
AUC	Area under the curve
BMI	Body mass index
CI	Confidence interval
cm	Centimeters
DBP	Diastolic blood pressure
DM	Diabetes mellitus
DR	Diabetic retinopathy
HbA1c	Glycated hemoglobin
HDL-C	High-density lipoprotein cholesterol
HR	Heart rate
kg	kilograms
LDL-C	Low-density lipoprotein cholesterol
LUHS	Lithuanian University of Health Sciences
NPDR	Non-proliferative diabetic retinopathy
OR	Odds ratio
PDR	Proliferative diabetic retinopathy
ROC	Receiver operating characteristic
SBP	Systolic blood pressure
SD	Standard deviation
TC	Total cholesterol
TG	Triglycerides
T1D	Type 1 diabetes
T2D	Type 2 diabetes
